# Cardiac Safety Assessment of Lazertinib: Findings From Patients With *EGFR* Mutation-Positive Advanced NSCLC and Preclinical Studies

**DOI:** 10.1016/j.jtocrr.2021.100224

**Published:** 2021-09-08

**Authors:** Seong Bok Jang, Kyeong Bae Kim, Sujin Sim, Byoung Chul Cho, Myung-Ju Ahn, Ji-Youn Han, Sang-We Kim, Ki Hyeong Lee, Eun Kyung Cho, Nahor Haddish-Berhane, Jaydeep Mehta, Se-Woong Oh

**Affiliations:** a Clinical Development Department, Yuhan Corporation, Seoul, Republic of Korea; b Yuhan R&D Institute, Yuhan Corporation, Yongin, Republic of Korea; c Division of Medical Oncology, Yonsei Cancer Center, Yonsei University College of Medicine, Seoul, Republic of Korea; d Division of Hematology-Oncology, Department of Medicine, Samsung Medical Center, Sungkyunkwan University School of Medicine, Seoul, Republic of Korea; e Center for Lung Cancer, Research Institute and Hospital, National Cancer Center, Goyang, Republic of Korea; f Department of Oncology, Asan Medical Center, University of Ulsan College of Medicine, Seoul, Republic of Korea; g Division of Medical Oncology, Department of Medicine, Chungbuk National University Hospital, Chungbuk National University College of Medicine, Cheongju, Republic of Korea; h Gil Medical Center, Gachon University College of Medicine, Incheon, Republic of Korea; i Janssen R&D, Spring House, Pennsylvania

**Keywords:** Non–small cell lung cancer, Tyrosine kinase inhibitor, Lazertinib, Cardiac toxicity

## Abstract

**Introduction:**

Lazertinib is a potent, irreversible, brain-penetrant, mutant-selective, and wild-type–sparing third-generation EGFR tyrosine kinase inhibitor (TKI), creating a wide therapeutic index. Cardiovascular adverse events (AEs), including QT prolongation, decreased left ventricular ejection fraction (LVEF), and heart failure, have emerged as potential AEs with certain EGFR TKI therapies.

**Methods:**

Cardiac safety of lazertinib was evaluated in TKI-tolerant adults with *EGFR* mutation-positive locally advanced or metastatic NSCLC receiving lazertinib (20–320 mg/d). QT intervals corrected with Fridericia’s formula (QTcF) prolongation, time-matched concentration-QTcF relationship, change of LVEF, and cardiac failure-associated AEs were evaluated. The clinical findings were supplemented by the following three preclinical studies: an in vitro hERG inhibition assay, an ex vivo isolated perfused rabbit heart study, and an in vivo telemetry-instrumented beagle dog study.

**Results:**

Preclinical evaluation revealed little to no physiological effect on the basis of electrocardiogram, electrophysiological, proarrhythmic, and hemodynamic parameters. Clinical evaluation of 181 patients revealed no clinically relevant QTcF prolongation by centralized electrocardiogram in any patient and at any dose level. The predicted magnitude of QTcF value increase at maximum steady-state plasma concentration for the therapeutic dose of lazertinib (240 mg/d) was 2.2 msec (upper bound of the two-sided 90% confidence interval: 3.6 msec). No patient had clinically relevant LVEF decrease (i.e., minimum postbaseline LVEF value of <50% and a maximum decrease in LVEF value from baseline of ≥10 percentage points). Cardiac failure-associated AE occurred in one patient (grade 2 decreased LVEF) and resolved without any dose modifications.

**Conclusions:**

Our first-in-human study, together with preclinical data, indicates that lazertinib is not associated with increased cardiac risk.

## Introduction

Cardiotoxicity and adverse events (AEs), such as QT interval corrected for heart rate (QTc) prolongation and decreased left ventricular ejection fraction (LVEF), have been associated with the use of various tyrosine kinase inhibitors (TKIs) in cancer treatment,^1^, ^2^, ^3^ including nilotinib, lapatinib, and osimertinib.^2^^,^^4^, ^5^, ^6^

QTc prolongation, a biomarker for increased risk of cardiac complications, is particularly important in safety assessments of TKIs owing to its association with potentially lethal arrhythmias, such as torsades de pointes, ventricular tachycardia, ventricular fibrillation, and sudden cardiac death.^7^, ^8^, ^9^ A QTc value of more than 500 msec or more than 60 msec increase in QTc value from baseline is considered a grade 3 AE and a clinically meaningful indicator of cardiac safety concern and possible cardiotoxicity.^1^^,^^10^^,^^11^

Decreased LVEF is a widely recognized risk factor for congestive heart failure and death.^12^, ^13^, ^14^ A clinically relevant decrease in LVEF has been defined as a LVEF decrease of at least 10 percentage points from baseline to an LVEF value of less than 50%.^15^

Third-generation EGFR TKIs (e.g., lazertinib and osimertinib) possess improved safety profiles compared with first- and second-generation EGFR TKIs.^16^^,^^17^ This is attributed to their selectivity for activating or T790M-mutant EGFR, while sparing wild-type EGFR.^16^^,^^18^ Nevertheless, osimertinib, which is indicated for treatment of *EGFR* mutation (*EGFR*m)-positive NSCLC,^17^^,^^19^ has been associated with increased risk of cardiac-related AEs, notably QTc prolongation and decreased LVEF.^6^ Because of these known risks of QTc prolongation, a QTc value of more than 500 msec has been specified as a condition for withholding, modifying the dose of, or permanently discontinuing osimertinib treatment depending on severity.^20^^,^^21^

Lazertinib (YH25448, JNJ-73841937; brand name: LECLAZA [Yuhan Corporation, Cheongju-si, Chungcheong buk-do, Republic of Korea]) is a potent, irreversible, brain-penetrant, mutant-selective, and wild-type–sparing third-generation EGFR TKI, creating a wide therapeutic index.^16^^,^^22^ As of January 2021, lazertinib 240 mg once-daily treatment has been approved in the Republic of Korea for patients with locally advanced or metastatic EGFR T790M mutation-positive NSCLC who have progressed on or after EGFR TKI therapy. Results from the first-in-human phase 1-2 study of the safety, tolerability, efficacy, and pharmacokinetics of lazertinib have been reported recently (NCT03046992).^16^

In this study, we report the findings on the cardiac safety of lazertinib at doses ranging from 20 to 320 mg/d in patients with *EGFR*m locally advanced or metastatic NSCLC who had previously received EGFR TKI treatment. The cardiac safety profile of lazertinib is supplemented by three preclinical studies of lazertinib, specifically an in vitro hERG inhibition assay, an ex vivo isolated perfused rabbit heart study, and an in vivo telemetry-instrumented male beagle dog study.

## Materials and Methods

### Preclinical Evaluation of Lazertinib

Detailed methodology of the in vitro hERG inhibition assay, ex vivo isolated perfused rabbit heart study, and in vivo telemetry-instrumented beagle dog study is available in the Supplementary Materials.

### Clinical Evaluation of Lazertinib

#### Study Design

This was an analysis of cardiac safety in patients participating in an ongoing phase 1-2 study of lazertinib for *EGFR*m NSCLC (NCT03046992). NCT03046992 is the first-in-human, open-label study of lazertinib in patients with EGFRm locally advanced or metastatic NSCLC who had previously received EGFR TKI treatment. The study design and findings have been described in detail elsewhere.^16^ The study protocol was approved by local institutional review board ethics committee at each site before study initiation.

The assessable population for this analysis consisted of patients receiving lazertinib doses ranging from 20 to 320 mg/d, from the dose escalation, dose expansion, and dose extension (second-line therapy cohort) phases of the study. We analyzed data from patients at 17 sites in the Republic of Korea up until data cutoff at September 30, 2019.

The study was conducted in accordance with the principles of the Declaration of Helsinki and the International Conference on Harmonization Good Clinical Practice. All patients, or their legally acceptable representatives, provided written informed consent before any study-related activities were undertaken.

#### Patient Eligibility

Adults aged 20 years or older with histologically or cytologically confirmed diagnosis of NSCLC with single activating *EGFR*m (L858R, exon 19 deletion, G719X, or L861Q) and who had baseline and postdose electrocardiogram (ECG) assessments (in triplicate) with time-matched plasma concentration data were eligible for the study. Key cardiovascular-related exclusion criteria included the following: mean resting QTc value of more than 470 msec obtained from three ECGs; LVEF less than 50%; any evidence of clinically active cardiovascular disease, defined as a history of symptomatic congestive heart failure or serious cardiac arrhythmia requiring treatment, or a history of myocardial infarction or unstable angina within 6 months from the start of the study; any clinically important abnormalities in rhythm, conduction, or morphology of resting ECG; and any factors that increase the risk of QTc prolongation or risk of arrhythmic events (e.g., heart failure, hypokalemia, congenital long QT syndrome). Other exclusion criteria are detailed in the publication of the primary study.^16^

#### Treatment

Patients received oral lazertinib at different dose levels (20 mg, 40 mg, 80 mg, 120 mg, 160 mg, 240 mg [therapeutic dose], and 320 mg) once daily and continuously in 21-day cycle until documented evidence of disease progression, unacceptable toxicity, noncompliance, withdrawal of consent, or investigator decision.

#### Cardiac Safety Outcome Measures

Cardiac-related AEs were monitored at baseline and at scheduled visits throughout the study (Supplementary Table 2) and graded according to Common Terminology Criteria for Adverse Events version 4.03.

Clinical ECG assessment and QT interval measurements were performed to evaluate QTc prolongation (measured by maximum postbaseline QT intervals corrected with Fridericia’s formula [QTcF] and maximum increase in QTcF from baseline) and to analyze time-matched concentration-QTcF relationship. LVEF was assessed using echocardiogram or multiple-gated acquisition (MUGA) scans at baseline and every 12 weeks.

##### ECGs and QT Interval Measurements

Resting 12-lead ECGs were measured and recorded in triplicate at approximately 2-minute intervals with the average value used for analysis. All ECG data were obtained after the patient had been resting semisupine for at least 10 minutes and were centrally analyzed. QT intervals, measured from the onset of the QRS complex to the end of the T wave and corrected for heart rate with Fridericia's correction formula, were determined and reviewed by an external cardiologist. Maximum postbaseline QTcF value and maximum increase in QTcF value from baseline were evaluated. QTc prolongation, defined as a maximum postbaseline QTcF value of more than 500 msec or a more than 60 msec maximum increase in QTcF value from baseline, was considered a dose-limiting or grade 3 toxicity that warranted the interruption of lazertinib treatment and conduct of regular ECGs until resolution to baseline.

##### Concentration-QTc Relationship Analysis

For concentration-QTc relationship analysis, time delay between change in QTcF value from baseline (ΔQTcF) and measured plasma concentration of lazertinib was first investigated by plotting ΔQTcF values over time. Because no time delay was observed, the relationship between ΔQTcF and plasma concentration was evaluated using a linear regression with ΔQTcF as the dependent variable and plasma concentration as the independent variable (Phoenix WinNonlin version 8.3; Certara, Princeton, NJ). After establishing linearity, a linear model was used to predict ΔQTcF with its corresponding two-sided 90% confidence interval (CI) at the maximum steady-state plasma concentration (C_max,ss_) for the therapeutic dosage of lazertinib (240 mg once daily).

##### Left Ventricular Ejection Fraction

LVEF was assessed using an echocardiogram or MUGA scan at baseline and every 12 weeks from the first dose (Supplementary Table 2). The specific modality of assessments (i.e., echocardiogram or MUGA scan) was kept consistent within a patient throughout the study, and patients were examined with the same operator and machine as far as possible.

Minimum postbaseline LVEF value and change in LVEF value from baseline were evaluated. In this study, a minimum postbaseline LVEF value of less than 50% and a maximum decrease in LVEF value from baseline of greater than or equal to 10 percentage points were used as cutoffs for determining clinically relevant decrease in LVEF.

##### Cardiac Failure-Associated AEs

The safety databases of Yuhan were searched for terms associated with cardiac failure or cardiomyopathy, using the Medical Dictionary for Regulatory Activities Preferred Terms of ejection fraction decreased, cardiac failure, chronic heart failure, and metabolic cardiomyopathy.

#### Statistical Analyses

Safety outcome measures evaluated in this study included QTc prolongation, LVEF, and other cardiac-related AEs. Patients who received at least one dose of lazertinib and had the appropriate ECG and LVEF data were analyzed. Subject demographics and all relevant safety outcome measures were summarized using descriptive statistics. Statistical Analysis System version 9.4 (SAS Institute Inc., Cary, NC) was used for all statistical analyses.

## Results

### Preclinical Evaluation of Lazertinib

All tested concentrations of lazertinib inhibited hERG currents by 25.5% to 59.8%, which were significantly higher than those of the vehicle control (2%, *p* < 0.01; Supplementary Fig. 1). The positive control, E-4031, inhibited hERG currents by 77.8% at 100 nM (*p* < 0.01 versus vehicle control). The mean (SEM) concentration that inhibits 50% (IC_50_) of lazertinib was 5.3 (2.0) μM.

In the isolated rabbit heart study, no relevant electrocardiographic, electrophysiological, and proarrhythmic changes were observed with lazertinib exposure of up to 30 μM at 750 msec (80 beats per min [bpm]), 500 msec (120 bpm), and 250 msec (240 bpm) cycle lengths (Supplementary Table 3). Only a slight prolongation in T_peak_T_end_ was noted with 30 μM lazertinib at 750 msec cycle length.

**Figure 1:**
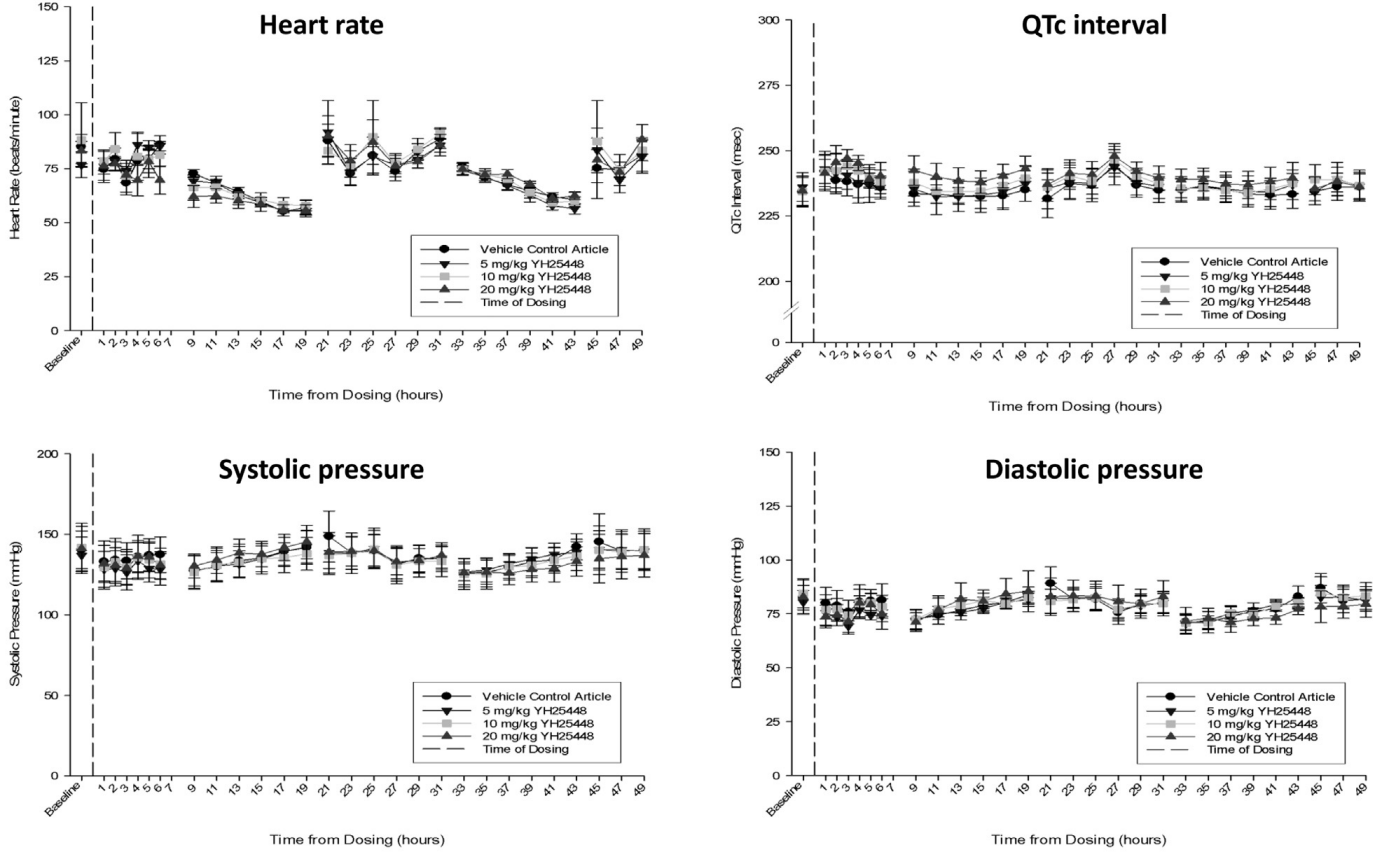
Cardiovascular parameters using telemetry monitoring after single oral administration of 5, 10, and 20 mg/kg of lazertinib in conscious male beagle dogs. Data are expressed as mean ± SEM (n = 4). QTc, QT interval corrected for heart rate.

No abnormal qualitative or quantitative ECG, hemodynamic, or body temperature measurements were observed after oral administration of up to 20 mg/kg lazertinib in dogs (Fig. 1 and Supplementary Fig. 2).

### Clinical Evaluation of Lazertinib

#### Patient Disposition, Baseline Characteristics, and Treatment Exposure

A total of 181 patients were analyzed. Patient demographics and baseline characteristics are detailed in Table 1. The median (range) age of the overall cohort was 62 (28–84) years. More than half of the patients (56.9%) were female, and all were Asian. More than half of the patients (56.4%) had one previous line of systemic therapy. The mean (SD) duration of therapy of the overall cohort was 11.3 (7.5) months.

**Table 1:** Patient Demographics and Baseline Characteristics

Characteristics	20 mg (n = 3)	40 mg (n = 27)	80 mg (n = 20)	120 mg (n = 25)	160 mg (n = 23)	240 mg (n = 78)	320 mg (n = 5)	Total (N = 181)
Age (y)								
Median (range)	58 (52–62)	61 (37–81)	67 (48–84)	63 (28–82)	62 (44–83)	62 (33–82)	64 (44–82)	62 (28–84)
Sex, n (%)								
Male	2 (66.7)	9 (33.3)	7 (35.0)	9 (36.0)	11 (47.8)	40 (51.3)	0	78 (43.1)
Female	1 (33.3)	18 (66.7)	13 (65.0)	16 (64.0)	12 (52.2)	38 (48.7)	5 (100.0)	103 (56.9)
Race, n (%)								
Asian	3 (100.0)	27 (100.0)	20 (100.0)	25 (100.0)	23 (100.0)	78 (100.0)	5 (100.0)	181 (100.0)
ECOG performance status, n (%)								
0	2 (66.7)	9 (33.3)	6 (30.0)	8 (32.0)	2 (8.7)	20 (25.6)	1 (20.0)	48 (26.5)
1	1 (33.3)	18 (66.7)	14 (70.0)	17 (68.0)	21 (91.3)	58 (74.4)	4 (80.0)	133 (73.5)
NSCLC classification by pathological characteristics, n (%)								
Adenocarcinoma	3 (100.0)	27 (100.0)	20 (100.0)	25 (100.0)	22 (95.7)	74 (94.9)	5 (100.0)	176 (97.2)
Adenosquamous carcinoma^a^	0	0	0	0	0	2 (2.6)	0	2 (1.1)
Other	0	0	0	0	1 (4.3)	2 (2.6)	0	3 (1.7)
NSCLC stage,^b^ n (%)								
IIIA	0	0	0	0	0	1 (1.3)	0	1 (0.6)
IIIB	0	0	1 (5.0)	1 (4.0)	1 (4.3)	2 (2.6)	0	5 (2.8)
IV	3 (100.0)	27 (100.0)	19 (95.0)	24 (96.0)	22 (95.7)	75 (96.2)	5 (100.0)	175 (96.7)
*EGFR* mutation result,^c^^*,*^^d^ n (%)								
Positive	3 (100.0)	27 (100.0)	18 (90.0)	25 (100.0)	23 (100.0)	77 (98.7)	4 (80.0)	177 (97.8)
L858R	1 (33.3)	6 (22.2)	9 (45.0)	11 (44.0)	11 (47.8)	23 (29.5)	2 (40.0)	63 (34.8)
Exon 19 deletion	2 (66.7)	21 (77.8)	9 (45.0)	14 (56.0)	12 (52.2)	53 (67.9)	2 (40.0)	113 (62.4)
Other	0	0	0	0	1 (4.3)	1 (1.3)	0	2 (1.2)
Negative	0	0	2 (10.0)	0	0	1 (1.3)	1 (20.0)	4 (2.2)
T790M mutation status*,*^c^^*,*^^e^ n (%)								
Positive	2 (66.7)	26 (96.3)	18 (90.0)	22 (88.0)	18 (78.3)	76 (97.4)	0	162 (89.5)
Negative	1 (33.3)	1 (3.7)	2 (10.0)	3 (12.0)	5 (21.7)	2 (2.6)	5 (100.0)	19 (10.5)
Previous lines of systemic therapy, n (%)								
1	3 (100.0)	16 (59.3)	7 (35.0)	14 (56.0)	11 (47.8)	51 (65.4)	0	102 (56.4)
≥2	0	11 (40.7)	13 (65.0)	11 (44.0)	12 (52.2)	27 (34.6)	5 (100.0)	79 (43.6)
Previous EGFR TKI treatment,^f^ n (%)								
Afatinib	0	1 (3.7)	3 (15.0)	3 (12.0)	6 (26.1)	28 (35.9)	1 (20.0)	42 (23.2)
Dacomitinib	0	0	0	1 (4.0)	0	0	0	1 (0.6)
Erlotinib	2 (66.7)	8 (29.6)	6 (30.0)	7 (28.0)	3 (13.0)	16 (20.5)	0	42 (23.2)
Gefitinib	1 (33.3)	19 (70.4)	12 (60.0)	16 (64.0)	16 (69.6)	40 (51.3)	4 (80.0)	108 (59.7)
Immediate previous EGFR TKI,^g^ n (%)								
Yes	3 (100.0)	21 (77.8)	14 (70.0)	18 (72.0)	15 (65.2)	71 (91.0)	1 (20.0)	143 (79.0)
<30 d	2 (66.7)	11 (52.4)	10 (71.4)	7 (38.9)	13 (86.7)	42 (59.2)	1 (100.0)	86 (60.1)
≥30 d	1 (33.3)	10 (47.6)	4 (28.6)	11 (61.1)	2 (13.3)	29 (40.8)	0	57 (39.9)
No	0	6 (22.2)	6 (30.0)	7 (28.0)	8 (34.8)	7 (9.0)	4 (80.0)	38 (21.0)
Time from end of last therapy to study entry (mo)^h^								
Median (range)	0.66 (0.6–1.9)	1.25 (0.3–4.2)	0.94 (0.3–4.9)	1.77 (0.3–23.2)	0.95 (0.3–8.0)	0.90 (0.3–7.1)	1.64 (0.6–12.6)	1.12 (0.3–23.2)

*Note:* Percentages were based on the number of patients in the respective dose cohorts.

AJCC, American Joint Committee on Cancer; ECOG, Eastern Cooperative Oncology Group; FFPE, formalin-fixed, paraffin-embedded; PCR, polymerase chain reaction; TKI, tyrosine kinase inhibitor.

a If intestinal differentiation component in lung adenocarcinoma is greater than 50%.

b According to the AJCC seventh edition.

c By central test.

d Multiple responses were allowed.

e The cobas *EGFR* mutation test version 2 (real-time PCR assay) is used for the qualitative detection and identification of mutations in exons 18, 19, 20 (including T790M), and 21 of the *EGFR* gene in DNA derived from FFPE tumor tissue from patients with NSCLC.

f All assessable patients received previous EGFR TKI treatment.

g Immediate previous EGFR TKI is defined as EGFR TKI taken as last regimen before the study entry with no subsequent therapy.

h Time from end of last therapy to study entry (mo) = (first date of study medication administration − end date of last therapy)/30.4375.

#### Cardiac Safety Assessment

##### QTc Prolongation

Maximum postbaseline QTcF values and maximum increase in QTcF values from baseline are presented in Table 2. On the basis of centralized ECG reading, no patient had a maximum postbaseline QTcF value of more than 500 msec or more than 60 msec maximum increase in QTcF value from baseline, suggesting there were no cases of clinically relevant QTc prolongation.

**Table 2:** Change in QTcF Values Assessed Using ECG in Patients Treated With Lazertinib as a Second-Line Therapy

Category	20 mg (n = 3)	40 mg (n = 27)	80 mg (n = 20)	120 mg (n = 25)	160 mg (n = 23)	240 mg (n = 78)	320 mg (n = 5)	Total (N = 181)
Maximum postbaseline QTcF value (msec), n (%)								
≤450	3 (100.0)	21 (77.8)	17 (85.0)	22 (88.0)	20 (87.0)	70 (89.7)	4 (80.0)	157 (86.7)
>450 to ≤480	0	5 (18.5)	2 (10.0)	3 (12.0)	3 (13.0)	8 (10.3)	1 (20.0)	22 (12.2)
>480 to ≤500	0	1 (3.7)	1 (5.0)	0	0	0	0	2 (1.1)
>500	0	0	0	0	0	0	0	0
Maximum increase in QTcF value from baseline (msec), n (%)^a^								
≤30	3 (100.0)	24 (88.9)	16 (80.0)	23 (92.0)	18 (78.3)	73 (93.6)	5 (100.0)	162 (89.5)
>30 to ≤60	0	2 (7.4)	3 (15.0)	2 (8.0)	5 (21.7)	5 (6.4)	0	17 (9.4)
>60	0	0	0	0	0	0	0	0

*Note:* Percentages were calculated using the number of patients in the safety analysis population for each treatment as the denominator and the number of patients with each event as the numerator.

ECG, electrocardiogram; QTcF, QT intervals corrected with Fridericia’s formula.

a Data were unavailable for one patient in each of the 40 mg and 80 mg dose groups; these patients did not have a baseline value that was confirmed by central assessment.

Most patients (86.7%) had a maximum postbaseline QTcF value of less than or equal to 450 msec, whereas 12.2% of the patients had a maximum postbaseline QTcF value of more than 450 msec to less than or equal to 480 msec. Maximum postbaseline QTcF values of more than 480 msec to less than or equal to 500 msec were observed in two patients (1.1%) (40 mg cohort: one patient [3.7%]; 80 mg cohort: one patient [5.0%]).

Most patients (89.5%) had a maximum increase in QTcF value of less than or equal to 30 msec from baseline, whereas 9.4% of the patients had a maximum increase in QTcF values of more than 30 msec to less than or equal to 60 msec from baseline. Across the dose cohorts, the highest proportion of patients who had a maximum increase in QTcF values of more than 30 to less than or equal to 60 msec from baseline was observed in the 160 mg cohort (21.7%).

QTcF prolongation was reported as a grade 1 AE in six patients (3.3%), as assessed by the investigator on the basis of local ECG reading. None of these AEs led to dose modification, and no obvious clinical symptoms of QTc prolongation were observed overall.

##### ΔQTcF Values With Time-Matched Concentrations of Lazertinib

**Figure 2:**
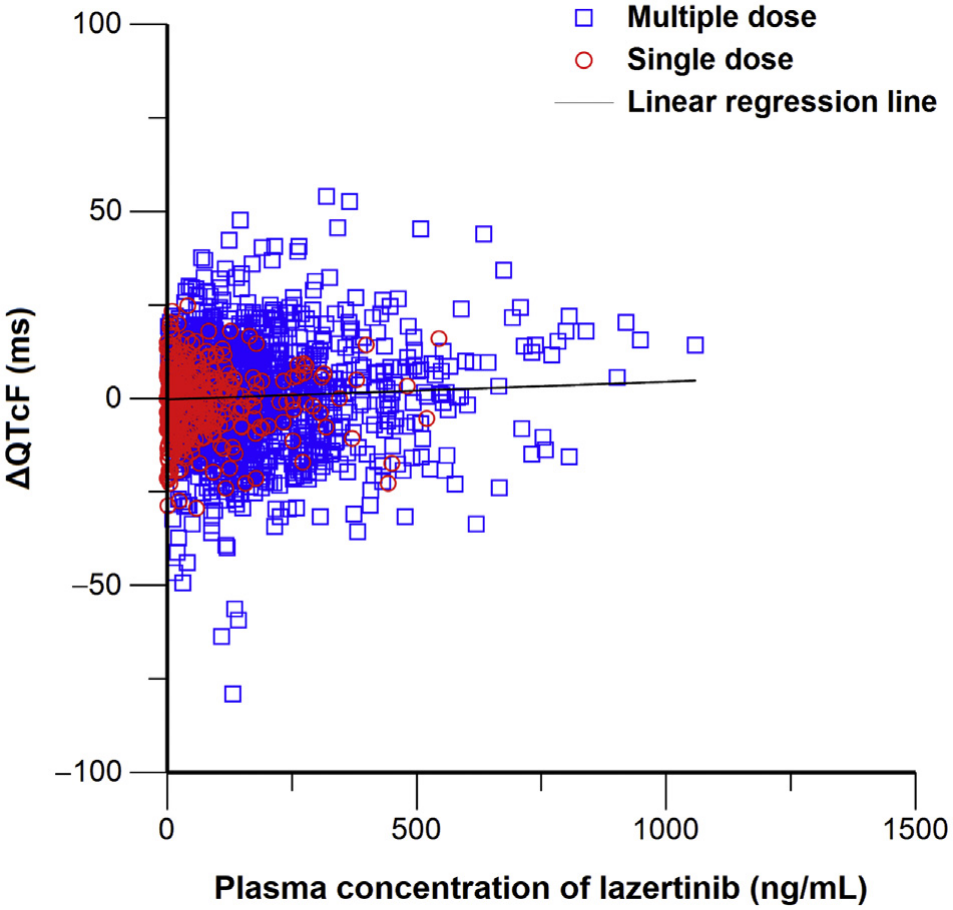
Plot of ΔQTcF values against time-matched concentrations of lazertinib. ΔQTcF, change in QT intervals corrected with Fridericia’s formula from baseline.

Considering T_max_ was two hours after single and multiple doses of lazertinib,^16^ no time delay between ΔQTcF and plasma concentration was observed (Supplementary Fig. 3). A total of 1852 ΔQTcF values with time-matched concentration were analyzed on the basis of linear regression (Fig. 2). A slope was estimated as 0.004735 msec/ng/mL with an intercept of −0.2686 msec (*p* = 0.0218). At the C_max,ss_ (517.15 ng/mL) of the therapeutic dose (240 mg) of lazertinib,^15^ the ΔQTcF was predicted to be 2.2 msec. The upper bound of the two-sided 90% CI for ΔQTcF was estimated to be 3.6 msec, which falls within the category of low concern (upper bound ≤5 msec), indicating there was low risk of QTc prolongation with lazertinib.

##### Left Ventricular Ejection Fraction

**Figure 3:**
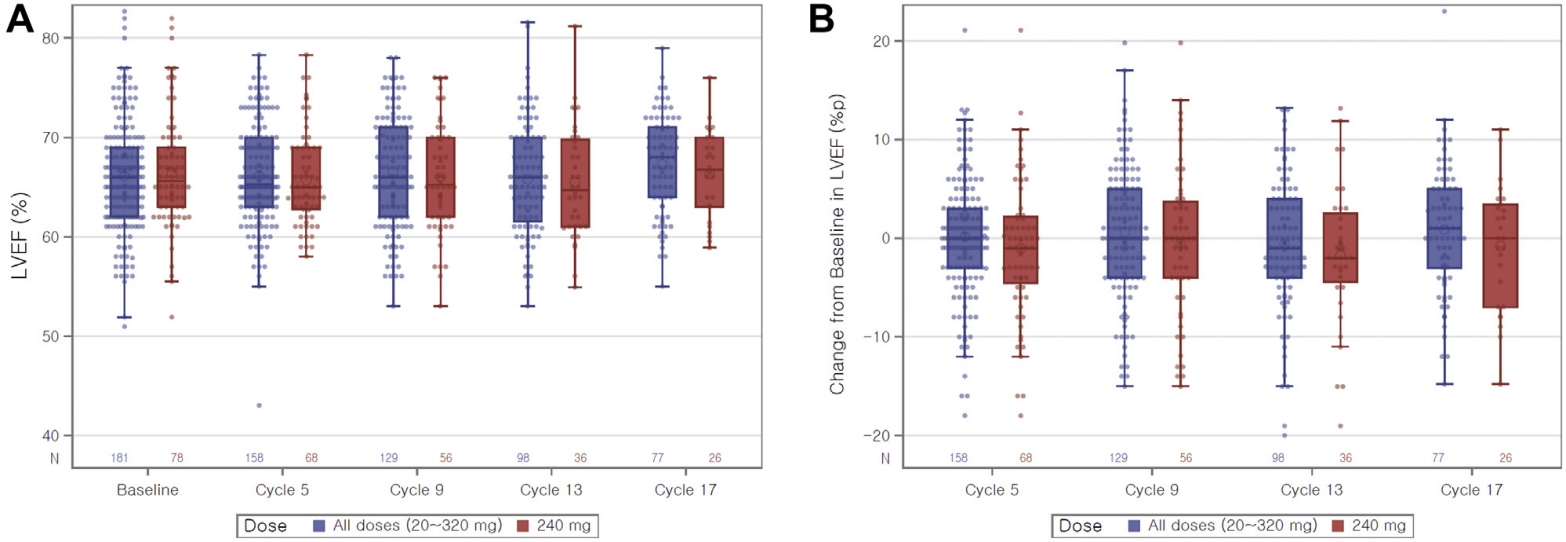
Distribution of LVEF values from echocardiogram or MUGA scan. (*A*) LVEF (%). (*B*) Change from baseline in LVEF (%p). %p, percentage point; LVEF, left ventricular ejection fraction; MUGA, multiple-gated acquisition.

There were no patients who had both a minimum postbaseline LVEF value of less than 50% together with a maximum decrease in LVEF value from baseline of greater than or equal to 10 percentage points (Table 3 and Fig. 3*A* and *B*).

**Table 3:** Assessment of LVEF Using Echocardiogram or MUGA Scan

Category	20 mg (n = 3)	40 mg (n = 27)	80 mg (n = 20)	120 mg (n = 25)	160 mg (n = 23)	240 mg (n = 78)	320 mg (n = 5)	Total (N = 181)
Minimum postbaseline LVEF value (%), n (%)^a^								
≥50	3 (100.0)	26 (96.3)	18 (90.0)	25 (100.0)	22 (95.7)	73 (93.6)	4 (80.0)	171 (94.5)
≥45 to <50	0	0	0	0	0	0	0	0
<45	0	0	1 (5.0)^b^	0	0	0	0	1 (0.6)
Maximum decrease in LVEF value from baseline (percentage point), n (%)^a^^*,*^^c^								
<10	3 (100.0)	21 (77.8)	14 (70.0)	24 (96.0)	19 (82.6)	61 (78.2)	4 (80.0)	146 (80.7)
≥10 to <15	0	4 (14.8)	3 (15.0)	1 (4.0)	3 (13.0)	9 (11.5)	0	20 (11.0)
≥15	0	1 (3.7)	2 (10.0)	0	0	3 (3.9)	0	6 (3.3)
Minimum postbaseline LVEF value of <50% and maximum decrease in LVEF value from baseline of ≥10 percentage points, n (%)	0	0	0	0	0	0	0	0

*Note:* Percentages were calculated using the number of patients in the safety analysis population for each treatment as the denominator and the number of patients with each event as the numerator.

Percentages were based on the number of patients in the respective dose cohorts.

The maximum decrease in LVEF value from baseline was calculated from the baseline LVEF value – the minimum postbaseline LVEF value.

AE, adverse event; LVEF, left ventricular ejection fraction; MUGA, multiple-gated acquisition.

a Data were unavailable for nine patients without postbaseline LVEF value.

b An abnormal change from baseline for one patient in which LVEF decreased from 51% to 43% but returned to 57% at follow-up without any dose modification. This was reported as an AE of “ejection fraction decreased” in cycle 5.

c None of the patients had a decrease in LVEF from baseline greater than 20 percentage points.

Most patients (94.5%) had a minimum postbaseline LVEF value of greater than or equal to 50%. Although, one patient (0.6%) from the 80 mg dose cohort was found to have a minimum postbaseline LVEF value of less than 45%, the LVEF value of this patient had decreased from 51% at baseline to 43% at cycle 5 but returned to 57% at the follow-up visit without any modification to the treatment dose. The incident was reported as a cardiac failure-associated AE (grade 2).

Most patients (80.7%) had a maximum decrease in LVEF value of less than 10 percentage points from baseline, whereas 11.0% of the patients had a maximum decline of 10 to less than 15 percentage points from baseline. Only six patients (3.3%) were found to have a maximum decrease in LVEF value of greater than or equal to 15 percentage points (one patient [3.7%] from the 40 mg cohort; two patients [10%] from the 80 mg cohort; three patients [3.9%] from the 240 mg cohort). Of these six patients, five had a history of cardiovascular risk factors (hypertension in two patients from the 240 mg cohort; old age [more than 70 y old] in three patients [two patients from the 80 mg cohort and one patient from the 240 mg cohort]; and cerebral infarction in one patient from the 80 mg cohort), and five patients had a high baseline LVEF of between 73% and 80% along with left ventricular concentric remodeling or diastolic dysfunction. None of these six patients had any cardiac failure-associated AEs.

#### Cardiac Failure-Associated AEs

Decreased LVEF occurred in one patient (0.6%) in the 80 mg dose cohort. The decreased LVEF was considered a drug-related, nonserious treatment-emergent AE (grade 2), which was resolved without any dose modification. No other cardiac failure-associated AEs were reported.

## Discussion

This study evaluated the preclinical cardiovascular effects of lazertinib and the cardiac safety of lazertinib treatment of 20 mg to 320 mg in patients with *EGFR*m locally advanced or metastatic NSCLC who had previously received EGFR TKI treatment. The key findings were as follows: (1) little to no physiological effect on the basis of ECG, electrophysiological, proarrhythmic, and hemodynamic parameters in preclinical studies; (2) no cases of clinically relevant QTc prolongation were observed with lazertinib by the centralized ECG reading in any patient and at any dose level; (3) no clinically relevant concentration-dependent QTc change was observed; the predicted magnitude of QTcF increase at the C_max,ss_ for the therapeutic dose was small and its upper bound was within the category of low concern; (4) a low proportion of patients met one or the other criteria for clinically relevant decrease in LVEF; no patient met both criteria; and (5) low rates of cardiac failure-associated AEs.

Our preclinical studies indicate a low risk of QTc prolongation, arrhythmias, or other physiological effects with lazertinib. In the hERG assay, lazertinib exhibited an IC_50_ of 5.3 μM that was associated with low risk of hERG inhibition and QTc prolongation.^23^ The IC_50_ value of lazertinib was 630-fold higher than the C_max,ss_ at the therapeutic dose of 240 mg after correction for unbound human concentration and was well above the minimum of 30-fold difference that would pose a risk of QTc prolongation or torsades de pointes.^24^

QTc prolongation is a known side effect of certain TKIs.^25^ Certain TKIs (e.g., lapatinib, imatinib, osimertinib) inhibit hERG potassium channels and cause a delay in cardiac repolarization, thus leading to QTc prolongation.^3^^,^^5^^,^^26^ In our study, QTc prolongation AEs as assessed by local ECG reading occurred in 3% of patients and were all of low grade (≤1). Maximum increase in QTcF value from baseline of more than 30 msec to less than or equal to 60 msec was observed in 17 patients (9.4%), all from the 40 mg to 240 mg cohort. The highest proportion of patients who had a maximum increase in QTcF values of more than 30 msec to less than or equal to 60 msec from baseline was observed in the 160 mg cohort (21.7%), whereas the proportions were 6.4% and 0% in the 240 mg and 320 mg cohorts, respectively. No patient had a maximum postbaseline QTcF value of more than 500 msec or more than 60 msec maximum increase in QTcF value from baseline on the basis of centralized ECG reading. The low risk of QTc prolongation with lazertinib was further revealed by analysis of ΔQTcF values with time-matched concentrations of lazertinib. In our study, the ΔQTcF from baseline at the C_max,ss_ for therapeutic dose was 2.2 msec with an estimated upper bound (two-sided 90% CI) of 3.6 msec, which was within the category of low concern.^21^ Collectively, there is an absence of clinically relevant QTc prolongation in our study.

Although our study included patients with cardiac risk factors (hypertension [38.7%], elderly patients ≥65 y old [38.7%], pulmonary embolism [2.2%], atrial fibrillation [1.1%], hypothyroidism [1.1%], and type 2 diabetes mellitus [0.6%]), no cases of clinically relevant QTc prolongation or decreases in LVEF were observed. These findings are consistent with the low incidence (0.6%) of cardiac failure-associated AEs. Nonetheless, given that our study excluded patients with more severe cardiovascular conditions (QTc value > 470 msec and LVEF < 50%), we acknowledge that our findings will need to be substantiated by further studies, including ongoing trials (NCT04248829; NCT04487080).

Third-generation TKIs, including lazertinib and osimertinib, have better selectivity for mutant EGFR over wild-type EGFR than earlier generations of TKIs.^16^^,^^27^ This increased selectivity has the potential to enhance the therapeutic index and reduce side effects, thereby improving the safety profile.^28^ Nevertheless, a recent analysis of the Food and Drug Administration Adverse Events Reporting System pharmacovigilance data indicates increased risk of cardiotoxicity with osimertinib compared with other TKIs (erlotinib, afatinib, gefitinib). The reporting OR for QT prolongation with osimertinib was 6.6 (95% CI: 3.4–12.8) relative to other EGFR TKIs.^29^ In randomized controlled trials, rates of cardiotoxicity were higher in the osimertinib than in the control treatment arms, and a pooled analysis of the AURA3 and FLAURA trials found that 3.9% of osimertinib-treated patients had both a minimum postbaseline LVEF value of less than 50% and a maximum decrease in LVEF value of greater than or equal to 10 percentage points from baseline.^26^ Taken together, these findings suggest that it is advisable to monitor for signs of cardiotoxicity in patients receiving EGFR TKIs, particularly osimertinib.

Several mechanisms for the cardiotoxicity of EGFR TKIs have been proposed. For example, decreased LVEF—and potentially QT prolongation—may be a result of inhibition of ErbB2 or HER2 and AMPK pathway, which causes reduced contractility, mitochondrial energy depletion, and cardiomyocyte apoptosis by means of Bcl-xL (B-cell lymphoma—extra large) or caspase 9-dependent pathways.^30^ Lazertinib exhibited 275-fold selectivity for HER2 over EGFR exon 19 deletion/T790M or L858R/T790M mutants that resulted in negligible inhibition of HER2, compared with a 6.7-fold selectivity for HER2 with osimertinib. Given this increased in vitro selectivity, lazertinib might be expected to have lower potential for cardiotoxicity than osimertinib or other TKIs that inhibit HER2. In our study, lazertinib had an IC_50_ of 5.3 μM in the hERG cellular patch-clamp assay, whereas an earlier study of osimertinib reported an IC_50_ of 0.57 μM in a similar assay.^31^ Our preclinical data thus support the idea that lazertinib might have reduced potential for HER2- or hERG-related cardiotoxicity, compared with other TKIs.

Overall, lazertinib had an acceptable cardiac safety profile. This first-in-human study of the cardiac safety of lazertinib provides an important and necessary initial confirmation of cardiac-related preclinical data. Nevertheless, this study had some limitations. The single-arm study design precluded direct comparisons of cardiac safety between lazertinib and other TKIs. Our study is considered to be in the early stages of clinical trials, hence, precluding further conclusions on the cardiac safety of lazertinib. In addition, given that only Asian patients from the Republic of Korea were analyzed in our study, our findings may not be generalizable to patients of different ethnicity. Nonetheless, our study presents important findings of the cardiac safety profile of lazertinib.

In conclusion, this study evaluated the cardiac safety of lazertinib 20 mg to 320 mg in Korean patients with *EGFR*m locally advanced or metastatic NSCLC who had previously received EGFR TKI treatment. Our first-in-human study together with preclinical data revealed that lazertinib is not associated with increased cardiac risk.

## CRediT Authorship Contribution Statement

**Seong Bok Jang:** Methodology, Formal analysis, Data curation.

**Kyeong Bae Kim, Sujin Sim:** Formal analysis, Data curation.

**Byoung Chul Cho, Myung-Ju Ahn, Ji-Youn Han, Sang-We Kim, Ki Hyeong Lee:** Methodology, Investigation, Data curation.

**Eun Kyung Cho:** Investigation, Data curation.

**Nahor Haddish-Berhane, Jaydeep Mehta, Se-Woong Oh:** Data curation.

## References

[1] Porta-Sánchez A., Gilbert C., Spears D. (2017). Incidence, diagnosis, and management of QT prolongation induced by cancer therapies: a systematic review. J Am Heart Assoc.

[2] Orphanos G.S., Ioannidis G.N., Ardavanis A.G. (2009). Cardiotoxicity induced by tyrosine kinase inhibitors. Acta Oncol.

[3] Jin Y., Xu Z., Yan H., He Q., Yang X., Luo P. (2020). A comprehensive review of clinical cardiotoxicity incidence of FDA-approved small-molecule kinase inhibitors. Front Pharmacol.

[4] Cirmi S., El Abd A., Letinier L., Navarra M., Salvo F. (2020). Cardiovascular toxicity of tyrosine kinase inhibitors used in chronic myeloid leukemia: an analysis of the FDA Adverse Event Reporting System database (FAERS). Cancers (Basel).

[5] Kloth J.S., Pagani A., Verboom M.C. (2015). Incidence and relevance of QTc-interval prolongation caused by tyrosine kinase inhibitors. Br J Cancer.

[6] Kunimasa K., Kamada R., Oka T. (2020). Cardiac adverse events in EGFR-mutated non-small cell lung cancer treated with osimertinib. JACC CardioOncol.

[7] Li M., Ramos L.G. (2017). Drug-induced QT prolongation and torsades de pointes. P T.

[8] Shah R.R., Morganroth J., Shah D.R. (2013). Cardiovascular safety of tyrosine kinase inhibitors: with a special focus on cardiac repolarisation (QT interval). Drug Saf.

[9] Strevel E.L., Ing D.J., Siu L.L. (2007). Molecularly targeted oncology therapeutics and prolongation of the QT interval. J Clin Oncol.

[10] Darpo B., Nebout T., Sager P.T. (2006). Clinical evaluation of QT/QTc prolongation and proarrhythmic potential for nonantiarrhythmic drugs: the International Conference on Harmonization of Technical Requirements for Registration of Pharmaceuticals for Human Use E14 guideline. J Clin Pharmacol.

[11] Brell J.M. (2010). Prolonged QTc interval in cancer therapeutic drug development: defining arrhythmic risk in malignancy. Prog Cardiovasc Dis.

[12] Wang T.J., Evans J.C., Benjamin E.J., Levy D., LeRoy E.C., Vasan R.S. (2003). Natural history of asymptomatic left ventricular systolic dysfunction in the community. Circulation.

[13] Ye Z., Lu H., Li L. (2018). Reduced left ventricular ejection fraction is a risk factor for in-hospital mortality in patients after percutaneous coronary intervention: a hospital-based survey. BioMed Res Int.

[14] Yoon G.J., Telli M.L., Kao D.P., Matsuda K.Y., Carlson R.W., Witteles R.M. (2010). Left ventricular dysfunction in patients receiving cardiotoxic cancer therapies are clinicians responding optimally?. J Am Coll Cardiol.

[15] U.S. Department of Health and Human Services, National Institutes of Health, National Cancer Institute Common Terminology Criteria for Adverse Events (CTCAE) version 4.0. https://evs.nci.nih.gov/ftp1/CTCAE/CTCAE_4.03/Archive/CTCAE_4.0_2009-05-29_QuickReference_8.5x11.pdf.

[16] Ahn M.J., Han J.Y., Lee K.H. (2019). Lazertinib in patients with EGFR mutation-positive advanced non-small-cell lung cancer: results from the dose escalation and dose expansion parts of a first-in-human, open-label, multicentre, phase 1–2 study. Lancet Oncol.

[17] Soria J.C., Ohe Y., Vansteenkiste J. (2018). Osimertinib in untreated EGFR-mutated advanced non-small-cell lung cancer. N Engl J Med.

[18] Shah R.R., Shah D.R. (2019). Safety and tolerability of epidermal growth factor receptor (EGFR) tyrosine kinase inhibitors in oncology. Drug Saf.

[19] Mok T.S., Wu Y.L., Ahn M.J. (2016). Osimertinib or platinum–pemetrexed in EGFR T790M–positive lung cancer. N Engl J Med.

[20] Food and Drug Administration Highlights of prescribing information—TAGRISSO (osimertinib) tablets, for oral use. https://www.accessdata.fda.gov/drugsatfda_docs/label/2020/208065s021lbl.pdf.

[21] Food and Drug Administration E14 clinical evaluation of QT/QTc interval prolongation and proarrhythmic potential for non-antiarrhythmic drugs. https://www.fda.gov/regulatory-information/search-fda-guidance-documents/e14-clinical-evaluation-qtqtc-interval-prolongation-and-proarrhythmic-potential-non-antiarrhythmic-0.

[22] Yun J., Hong M.H., Kim S.Y. (2019). YH25448, an irreversible EGFR-TKI with potent intracranial activity in EGFR mutant non-small cell lung cancer. Clin Cancer Res.

[23] Gintant G.A., Su Z., Martin R.L., Cox B.F. (2006). Utility of hERG assays as surrogate markers of delayed cardiac repolarization and QT safety. Toxicol Pathol.

[24] Redfern W.S., Carlsson L., Davis A.S. (2003). Relationships between preclinical cardiac electrophysiology, clinical QT interval prolongation and torsade de pointes for a broad range of drugs: evidence for a provisional safety margin in drug development. Cardiovasc Res.

[25] Chen M.H., Kerkelä R., Force T. (2008). Mechanisms of cardiac dysfunction associated with tyrosine kinase inhibitor cancer therapeutics. Circulation.

[26] Ewer M.S., Tekumalla S.H., Walding A., Atuah K.N. (2021). Cardiac safety of osimertinib: a review of data. J Clin Oncol.

[27] Tan A.C., Teh Y.L., Lai G.G.Y., Tan D.S.W. (2019). Third generation EGFR TKI landscape for metastatic EGFR mutant non-small cell lung cancer (NSCLC). Expert Rev Anticancer Ther.

[28] Rho J.K., Lee I.Y., Choi Y.J. (2017). Superior efficacy and selectivity of novel small-molecule kinase inhibitors of T790M-mutant EGFR in preclinical models of lung cancer. Cancer Res.

[29] Anand K., Ensor J., Trachtenberg B., Bernicker E.H. (2019). Osimertinib-induced cardiotoxicity: a retrospective review of the FDA Adverse Events Reporting System (FAERS). JACC CardioOncol.

[30] Chaar M., Kamta J., Ait-Oudhia S. (2018). Mechanisms, monitoring, and management of tyrosine kinase inhibitors-associated cardiovascular toxicities. Onco Targets Ther.

[31] Jin T., Hu B., Chen S. (2018). An in vitro assay of hERG K+ channel potency for a new EGFR inhibitor FHND004. Front Pharmacol.

