# Supplementary material for: Cardiac Safety Assessment of Lazertinib: Findings From Patients With EGFR Mutation-Positive Advanced NSCLC and Preclinical Studies
Source: JTO Clin Res Rep. 2021 Sep 8;2(10):100224. doi: 10.1016/j.jtocrr.2021.100224 (PMC8501499; doi:10.1016/j.jtocrr.2021.100224)
Supplement: Supplementary material [file mmc1.docx]

**SUPPLEMENTARY MATERIAL**

**Supplementary Methods**

**In vitro *hERG inhibition assay***

Cell line and Reagents

Chinese hamster ovary (CHO) cells transfected with hERG cDNA were obtained from bSys GmBH (Witterswill, Switzerland) and cultured in reconstituted 90% Dulbecco’s Modified Eagle’s Medium Nutrient Mixture F-12 supplemented with 9% Fetal Bovine Serum, 0.9% Penicillin/streptomycin and 0.1% 50 µg/mL Hygromycin B.

CHO cells were cryopreserved as a 0.9 mL cell suspension containing 0.1 mL of DMSO. At least 7 days prior to use, a vial of frozen cells was thawed and cultured and the absence of mycoplasma contamination was verified. Cells were incubated at 37ºC in a 5% CO_2_ atmosphere as a monolayer in 75 cm^2^ culture flasks and then sub-cultured twice-weekly using 0.05% trypsin-EDTA (Lot #1715699, #1748048). Mycoplasma testing was performed on the date of cell seeding.

Electrophysiology

***Preparation of solutions for electrophysiological experiments***

The extracellular solution was a Normal Tyrode solution adjusted to pH 7.39–7.40 using 3 M NaOH and contained 143 mM NaCl, 5.4 mM KCl, 5.0 mM HEPES, 0.33 mM NaH_2_PO_4_, 0.5 mM MgCl_2_, 10.0 mM glucose, and 1.8 mM CaCl_2_. Stock solution of the Normal Tyrode solution (10x) was prepared without glucose and CaCl_2_, and stored refrigerated [Archives No.: Korea Institute of Toxicology (KIT) 14002(18)]. On the day of treatment, the stock solution was diluted 10-fold with distilled water. Glucose and CaCl_2_ were added and the resultant osmolarity was 299 mOsm. Normal Tyrode solution was held at room temperature during the experiment and any unused solution was discarded at the end of treatment. The internal solution contained 130 mM KCl, 1.0 mM MgCl_2_, 5.0 mM EGTA, 5.0 mM MgATP, and 10.0 mM HEPES and was adjusted to approximately pH 7.29 using 3 M KO. Internal solution was stored frozen as 1 mL aliquots [Archives No.: KIT-14002(18)], and thawed for use as required.

***Application***

Cells were placed in an application chamber and initially superfused by gravity with Normal Tyrode solution (Sigma-Aldrich) at a flow rate of approximately 5 mL/min for approximately 3 minutes. Test solutions were subsequently superfused for approximately 10 minutes at the same flow rate to achieve steady-state concentration.

***Whole-cell patch-clamp***

hERG potassium channel current across the cell membrane was recorded under voltage-clamp conditions using the whole-cell patch clamp technique. Cells were trypsinized (0.05% trypsin-EDTA (Lot #1715699, #1748048), placed in the bath chamber and then allowed to settle for approximately 20 minutes. The bath chamber was filled with the internal solution and the electrode inserted into its holder. The electrode tip was attached to the cell membrane using a micromanipulator.

Voltage clamp mode was selected on the Axopatch 200B (Axon Instrument, USA) and negative pressure was applied to form a giga-seal to rupture the cell membrane and achieve whole-cell status. After whole-cell mode, the membrane potential of the cell was held at -80 mV and the cells hyperpolarized for 100 ms to -90 mV then depolarized for 2 s to +20 mV followed by a 3 s repolarization to -40 mV for hERG potassium channel current evaluation. The pulse interval used was approximately 20 seconds and the temperature of the Normal Tyrode solution in the application chamber was monitored and maintained at approximately 37ºC (actual values ranged from 36.7 to 37.2ºC) using a TC-344B controller (Warner Instruments, USA).

Data was acquired using pipette electrodes fabricated from borosilicate-glass capillaries (Harvard Apparatus Ltd., UK) using a PP-830 puller (Narishige Scientific Instrument Lab., Japan). When filled with the pipette solution, electrical resistance of the electrodes ranged from 3.1 to 4.1 MΩ.

***Treatment with test solutions***

A total of 19 cells were treated with the vehicle control (0.3% dimethyl sulfoxide [DMSO] in Normal Tyrode solution), positive control (100 nM E-4031, Sigma-Aldrich) or lazertinib at increasing concentrations of 1, 2.5, 5.0 and 10.0 µM.

***Statistical analysis***

Half-maximal inhibitory concentration (IC_50_) was calculated using the Hill equation: *f=xH/(IC_50_H+xH)*; where *H* is the Hill coefficient, *x* is the concentration, and *f* is the inhibition ratio. Test item effects on the repolarizing potassium channel current (Ikr) were presented as percentage inhibition at each concentration. Data were analyzed for homogeneity of variance using Bartlett's test. Homogeneous data were analyzed using Analysis of Variance (ANOVA) and significant inter-group differences were analyzed using Dunnett's test. Student’s t-test was used to determine differences between the positive control group and the vehicle control. Statistical analysis was performed by using (SAS/STAT Version 9.2, Cary, USA).

***Effects of lazertinib on electrocardiographic, electrophysiological, and proarrhythmic indices in isolated perfused rabbit hearts***

Tissues and Reagents

Female New Zealand White rabbits (n=4) were anesthetized. Hearts were surgically removed and stored in cold Krebs-Henseleit buffer containing 118 mM NaCl, 4 mM KCl, 22 mM NaHCO_3_, 1.1 mM MgCl_2_, 0.4 mM NaH_2_PO_4_, 1.8 mM CaCl_2_, 5 mM dextrose, 2 mM pyruvate and 0.038 mM creatine.

Preparation of hearts for experiment

Detailed experimental methodology has been published previously.[^33^](#_ENREF_33) The aorta was cannulated and stimulating electrodes were sutured to each side of the distal bundle of His. Hearts were perfused at a constant pressure of 80 cm H_2_O with Krebs-Henseleit solution at 33–35°C. The perfusate was equilibrated with 95% O_2_ and approximately 5% CO_2_ to maintain the pH at 7.35. A monophasic action potential (MAP) recording electrode was inserted into the left ventricle and subendocardium, which is rich in Purkinje fibers. Another MAP recording electrode and a reference electrode were placed on the left ventricular epicardium. The reference electrode was perfused at about 1 ml/min with isotonic KCl, enriched with 1.8mM CaCl_2_, and grounded. Human intervention ceased after the heart was mounted on a Langendorff aparatus to allow for conduct and analysis of the experiment.

Hearts were stimulated at 1.5 times threshold stimulation. The preparation was stimulated until instability of MAP duration (MAPD; determined by the Best Easy Systematic [BES] method) of the last 20 consecutive MAP trains became <10 ms, if the following conditions were met: automaticity and escape cycle length >1000 ms, threshold stimulation current <300 µA, coronary perfusion >17 ml/min, ectopic rate <40 ectopics for a 10-minute interval, the cardiac activation time <60ms, and the product of the number of ectopic beats × instability ((beats/min)ms) <750 ms. Preparations that did not meet these conditions were rejected.

Experimental protocol

The experiment was conducted in two phases – a small protocol and a large protocol. In the small protocol, hearts were equilibrated every minute at every concentration of lazertinib (0, 1, 3, 10, 30 µM) for approximately 15 minutes. Hearts were subjected to MAPs of a 10-beat train at 1000 and 300 ms were recorded together with a train of 30 MAPs stimulated at a cycle length of 1000 ms. On odd-numbered minutes a 30-second MAP train with alternating stimulation intervals of long and short sequences was stimulated: five cycles at 1 second, followed by an irregular sequence: 0.4, 1.8, 1, 0.75, 0.45, 0.6, 2, 0.45, 0.45, 2.6, 2, 0.5, 0.9, 0.9, 1.2, 0.3, 0.3, 0.3, 1.1 and 2 seconds and terminated by another five cycles at 1 second.

In the large protocol, following equilibration, baseline measurements were obtained and hearts were exposed to increasing concentrations of lazertinib for 20 minutes at each concentration. Hearts were paced at 80 bpm for the first 14 minutes of each 20‑minute period, while during the last 6 minutes hearts were paced at 120 and 240 bpm for 3 minutes at each cycle length (750, 500 and 250 ms). The QRS duration, QT interval, QT_peak_ interval, T_peak_-T_end_ duration and the appearance of early after depolarizations, as well as MAP duration at 30%, 50%, and 90% of repolarization (MAPD_30_, MAPD_50_ and MAPD_90_, respectively) along with beat-to-beat variability (BVR) of the MAPD_90_ were also determined.

Electrophysiology

Electrophysiological measurements were recording using the SCREENIT system. MAPs were digitized at 1 kHz (12-bit resolution) and saved to a disk. The MAP upstroke was sampled at 10 kHz. Amplitudes of MAPs were required to be 44mV and exceeding 450% of the average upstroke in a MAP train to be considered valid. MAPD_10–90_ were measured from the midpoint of the upstroke until 10, 20,…, 90% repolarization. A 10% increase in the average MAPD_60_ was considered a significant prolongation. Triangulation was measured as the repolarization time from MAPD_30_ to MAPD_90_. Triangulation was considered significant if the change in repolarization time was 429 ms.

Reverse frequency dependence was measured as the difference between the MAPD_60_ of the first 10 and that of the last 20 MAPs of a 30-pulse train at 1Hz. A difference >6 ms as compared to that in drug-free solution was considered as significant frequency-dependence. Any MAPs where the upstroke was not within 80 ms after the stimulus was considered an ectopic beat. Repolarization disturbances recorded from a MAP electrode result from ectopic beats or differences in cellular repolarization times at local or remote sites from the MAP electrode. The number of ectopics was reported as an average (ectopics/10 minute) during the last 3 minutes of any 10–15 minute perfusion. A positive voltage deflection interrupting late phase 2 or phase 3 of MAP repolarization was regarded as an early after-depolarization (EAD).

Ventricular tachycardia was defined as >6 consecutive beats with similar appearance. TdP was identified when >3 beats with positive and negative deflections appeared on the MAP, and where the MAP amplitude and upstroke were variable, but oscillated with a predictable pattern, similar to that seen as prototypical twisting of the ECG around the isoelectric line. Ventricular fibrillation was defined when consecutive beats appeared random with no reproducible pattern. If proarrhythmic activity became too great the computer automatically terminated the experiment. These experiments were nevertheless included in the analysis up to the point of excessive proarrhythmic activity and automatic termination, and the particular proarrhythmia was noted.

Data analysis

Data were recorded by a 12-bit analogue to digital converter sampling each of the two channels: subendocardial and epicardial MAPs. Data were analyzed beat by beat during the experiment, compressed and saved to disk, and stored in an ASCII format.

MAPD_60_ instability was assessed using a nonparametric test based on the assumption that MAPD_60_ distribution would be inconsistent during drug perfusion. The BES method was used to estimate the MAPD_60_ to minimize bias resulting from a few exceptionally long or short MAPDs. Steady-state MAPs were categorized according to their MAPD_60_ and the median, upper 25% and lower 25% values were computed by linear interpolation. An instability value was obtained by computing the difference between the upper and lower quartile estimates in milliseconds. An instability value >14 ms was considered significant. For the experimental MAP trains in each drug concentration, the last 20 MAPs for the final 3 minutes of drug perfusion in the short protocol were used, amounting to a total of 60 MAPs. A difference >14 ms between control and drug effects was considered significant.

***Effects of lazertinib on cardiovascular function parameters in telemetry-instrumented dogs***

Animals

All procedures involving animals were approved by the committee on laboratory animal use and conducted in accordance with a protocol approved by the Institutional Animal Care and Use Committee in Association for Assessment and Accreditation of Laboratory Animal Care-accredited facilities.

Dogs have historically been used in safety evaluation studies and are accepted by the appropriate regulatory agencies. The oral route of administration was selected because it is the intended route of administration in humans.

Three naïve and five non-naïve male purebred beagle dogs (Covance Research Products Inc., Cumberland, Virginia) were prequalified for this study and were acclimated to the study room for 2 weeks prior to Day 1 of the dosing phase. Animals were acclimated to oral gavage dosing with 5 mL/kg of reverse osmosis (RO) water twice during the predose phase. At initiation of dosing, animals were aged 18–22 months old with body weights between 10.6–12.8 kg. Animals were co-housed in stainless steel cages, except during cardiovascular monitoring periods (within 4 hours before the beginning and after the end of telemetry collections), when animals were individually housed. Water and certified canine diet #5007 (PMI Nutrition International Certified LabDiet^®^) were provided *ad libitum*.

Animals were checked twice daily (a.m. and p.m.) for mortality, abnormalities, and signs of pain or distress, except on days of transfer on and off study and on Days 2 and 14 of the predose phase. Abnormal findings were recorded. Cageside observations were conducted once daily, except on days when detailed observations were conducted (i.e. three times during the predose phase).

Surgical implantation of PCT transmitters with intravenous or diaphragmatic ECG leads

An ECG, blood pressure, and body temperature transmitter (Data Science International [DSI]; Model TL11M2 D70 PCT) was implanted into the abdomen of the dog and sutured to the abdominal wall, in accordance with Covance standard operating procedures under a separate study number and at least 2 weeks prior to study initiation. The ECG leads of the transmitter were arranged in an approximate Lead II configuration. The negative ECG lead was placed into the right jugular vein and advanced towards the heart. The positive lead was sutured to the abdominal side of the diaphragm close to the apex of the heart. The pressure catheter was placed in the aorta via the femoral artery to assess aortic pressure.

Study design and treatment

Two naïve and two non-naïve animals were selected by the Study Director based on acclimation data during the predose phase and assigned to the study using a computerized procedure. Animals selected for the study were dosed according to a Latin square design ([Supplementary Table 1](#_Supplementary_Table_1._1)). Control of bias was based on the use of a Latin square design. The dosing regimen was designed to ensure all dose levels were represented on each dosing day, each animal was administered each dose only once, and every animal was administered a unique dose sequence. Each animal was administered all doses. Animals were not separated into dose groups; however, due to software requirements, arbitrary group assignments were made.

The rationale for the dosing design was based on a previous dose-range-finding and 14-day repeat-dose toxicity study in beagle dogs. Oral administration of 30 mg/kg was tolerated in the dose-range-finding phase in one male and one female beagle dog, whereas 60 and 90 mg/kg were not tolerated, resulting in the early sacrifice of the male and female on Days 5 and 9, respectively. In the 14-day repeat-dose toxicity study, 15 mg/kg was tolerated, whereas 30 and 50 mg/kg were not tolerated, resulting in the early sacrifice on Day 7 of all animals administered 50 mg/kg and of one female administered 30 mg/kg. Based on these results, the high dose of 20 mg/kg was selected for the present dog safety pharmacology cardiovascular study. It was expected that oral administration of a single dose of this level would be well tolerated in male beagles. The low and mid doses of 5 and 10 mg/kg were intended to provide a range of exposure to assess the potential effects of the test article on cardiovascular function.

Each animal was administered all doses of vehicle control (Covance Research Products Inc., Cumberland, Virginia) and test articles (lazertinib; Yuhan R&D Institute, Republic of Korea) on Days 1, 8, 15 and 22. The vehicle control article contained 0.5% (w/v) methylcellulose (1500 centipoise) and 50 mM citric acid in reverse osmosis water (pH adjusted to 3.0–3.2).

Telemetry

Telemetry data (ECG, hemodynamic and body temperature measurements; DSI Dataquest^®^ OpenART^®^) were recorded continuously for at least 22 hours before review by the Study Director to determine if the animal was qualified for the study. For each dosing day, ECG, arterial pressure, and body temperature measurements were recorded for at least 90 minutes prior to dosing and continuously through at least 49 hours after dosing. Data collection was paused briefly (<30 minutes) after 25 hours postdose for transfer of data to a network drive. Data collection resumed following electronic data transfer.

Analysis of telemetry data

Telemetry data generated by Ponemah (P3P [Ponemah Physiology Platform]) analysis system were analyzed at baseline (1 h-segment average prior to dosing) and postdose (1 h-segment averages during first light phase, 2 h-segment averages during first dark phase, 2 h-segment averages during second light/dark phases and 2 h-segment averages during third light phase).

***Qualitative ECG evaluation***

For qualitative ECG evaluation, 1 to 1.5 minutes of continuous ECG recordings for each animal were isolated prior to dosing and 2, 4, 8, 12, 18, 24, 36 and 48 hours postdose. Data segments were evaluated for abnormal waveform morphology and arrhythmias within 10 minutes of the predetermined time points.

***Quantitative ECG evaluation***

ECG segments collected during the dosing phase were used to construct a library of representative ECG waveforms for each animal. The library for each animal was approved by a qualified expert and used to quantify ECG waveforms from that individual animal.

ECG waveforms collected during the dosing phase matching the library were analyzed to determine PR and QT intervals, and QRS duration. The corrected QT (QTc) interval was determined using an individual animal correction factor (IACF). The RR interval and the heart rate derived (HR_RR_) from the ECG waveform were determined for use in QT interval correction only.

For each animal, QTc interval values were calculated using an IACF based on data collected on the day of the control dose for that animal and applied to all doses for the same animal. An IACF was generated for each animal by fitting a linear regression line to 1‑minute means of QT versus HR_RR_. The QTc interval was statistically analyzed and reported. Individual animal QTc values were calculated for each 1‑minute interval according to the formula *QTc = QT ‑ IACF x (HR_RR_ - 75)*, where HR_RR_ is denoted in bpm.

***Hemodynamic and body temperature measurements***

Hemodynamic parameters (heart rate derived from the arterial pressure waveform; systolic, diastolic, and mean arterial pressures; and arterial pulse pressure) and body temperature were analyzed.

Statistical analysis

Postdose telemetry data (with the exception of RR interval and HR_RR_) were analyzed using a modified Latin square design repeated measures analysis of covariance (model 1: response = baseline + treatment + time + treatment * time + animal + day repeated time / subject = animal * day), with the mean baseline observation for each subject on each dosing day as the covariate. Results were reported as significant at the 5% or 1% significance level, as applicable. The covariate-adjusted mean, arithmetic mean, standard deviation, and sample size for each group were reported at each time point for all applicable parameters.

**Supplementary Tables and Figures**

**Supplementary Table 1.** Latin square dosing design for the in vivo telemetry-instrumented male beagle dog study

| **Animal identification^a^** | **Day 1** | **Day 8** | **Day 15** | **Day 22** |
| --- | --- | --- | --- | --- |
| H13901 | Low | Control^b^ | High | Mid |
| H13902 | Mid | High | Control | Low |
| H13903 | High | Low | Mid | Control |
| H13904 | Control | Mid | Low | High |
| ^a^ Animals were dosed in ascending order based on dose level  ^b^ Control animals were administered the vehicle control article containing 0.5% (w/v) methylcellulose (1500 centipoise) and 50 mM citric acid in reverse osmosis water (pH adjusted to 3.0–3.2)  Control: 0 mg/kg (dose level); 5 mL/kg (dose volume); 0 mg/mL (dose concentration)  Low: 5 mg/kg; 5 mL/kg; 1 mg/mL  Mid: 10 mg/kg; 5 mL/kg; 2 mg/mL  High: 20 mg/kg; 5 mL/kg; 4 mg/mL | | | | |

**Supplementary Table 2.** Planned schedule for cardiac safety assessments

| **Part A, Dose escalation phase** | | | | | | | | | | | | |
| --- | --- | --- | --- | --- | --- | --- | --- | --- | --- | --- | --- | --- |
| **Activities** | **Screening** | **Single Dose/ Cycle 0**  **(7±2 day Cycle)** | | | **Multiple Dose/ Cycle 1**  **(21 day Cycle)^a^** | | | **Cycle 2** | **Cycle 3 onward^b^** | **Unscheduled Visit^c^** | **Discontinuation^d^** | **28 day follow-up^e^** |
| **Visit** | **1** | **2** | **3** | **4** | **5** | **6** | **7** | **8** | **9 onward** |  |  |  |
| **Day** | **-28 to -1** | **D1** | **D2** | **D3** | **D1** | **D8** | **D15** | **D1** | **D1** |  |  |  |
| **Study Visit Window  (Days)** | **N/A** | **0** | **0** | **0** | **0** | **±1** | **±3** | **0** | **±7** | **N/A** | **+7** | **+7** |
| 12-lead ECG^f^ | X | X | X | X | X | X | X | X | X | (X) | X |  |
| Echocardiography/ MUGA^g^ | X |  |  |  | 12-weekly relative to first dose of multiple dosing | | | | | (X) | X |  |
| Cardiovascular-related AEs^h^ | X | X | X | X | X | X | X | X | X | X | X | X |
| Laboratory test: PK blood sampling^j,k^ |  | X | X | X | X | X | X | X |  |  |  |  |
| **Part B, Dose expansion phase** | | | | | | | | | | | | |
| **Activities** | **Screening** | - | - | - | **Multiple Dose/ Cycle 1**  **(21 day Cycle)^a^** | | | **Cycle 2 onward^2^** | - | **Unscheduled Visit^c^** | **Discontinuation^d^** | **28 day follow-up^e^** |
| **Visit** | **1** | - | - | - | **2** | **3** | **4** | **5 onward** | - |  |  |  |
| **Day** | **-28 to -1** | - | - | - | **D1** | **D8** | **D15** | **D1** | - | **N/A** | **+7** | **+7** |
| **Study Visit Window (Days)** | **0** | - | - | - | **0** | **±3** | **±3** | **0^km^, ±7^n^** | - |  |  |  |
| 12-lead ECG^f^ | X | - | - | - | X | X | X | X | - | (X) | X |  |
| Echocardiography/ MUGA^g^ | X | - | - | - | 12-weekly relative to first dose | | | | - | (X) | X |  |
| Cardiovascular-related AEs^h^ | X | - | - | - | X | X | X | X | - | X | X | X |
| Laboratory test: PK blood sampling^j,k^ |  |  |  |  | X | X | X | X (D1–D2 Cycle 2 only) |  |  |  |  |
| **Part C, Dose extension phase** | | | | | | | | | | | | |
| **Activities** | **Screening** | - | - | - | **Multiple Dose/ Cycle 1**  **(21 day Cycle)^a^** | | | **Cycle 2** | **Cycle 3 onward^b^** | **Unscheduled Visit^c^** | **Discontinuation^4^** | **28 day follow-up^e^** |
| **Visit** | **1** | - | - | - | **2** | **3** | **4** | **5** | **6 onward** |  |  |  |
| **Day** | **-28 to -1** | - | - | - | **D1** | **D8** | **D15** | **D1** | **D1** |  |  |  |
| **Study Visit Window**  **(Days)** | **0** | - | - | - | **0** | **±3** | **+7** | **±7** | **±7** | **N/A** | **+7** |  |
| 12-lead ECG^f^ | X | - | - | - | X | X | X | X | X | (X) | X |  |
| Echocardiography/ MUGA^g^ | X | - | - | - | 12-weekly relative to first dose | | | | | | X |  |
| Cardiovascular-related AEs^h^ | X | - | - | - | X | X | X | X | X | X | X | X |
| Laboratory test: PK blood sampling^j,k^ |  |  |  |  | X | X | X |  |  |  |  |  |
| Notes: Assessments that were not mandatory for all patients were bracketed i.e. (X)  ^a^ A treatment cycle was defined as 21 days for the purposes of scheduling procedures and evaluations. There was no scheduled break between cycles. ^b^ Study visits occurred on Day 1 of every cycle until Cycle 7 and every other cycle from Cycle 7 onward for dose escalation and dose expansion. But study visits for dose extension occurred on Day 1 of every other cycle from Cycle 3 onward (C1D1, C2D1, C3D1, C5D1, C7D1…).  ^c^ Unscheduled visits was arranged if necessary. Study procedures were at the discretion of the investigator.  ^d^ All patients attended a discontinuation visit within 7 days of permanent discontinuation of study drug, where all procedures for the discontinuation visit were performed. The discontinuation visit occurred before the start of a new treatment and the reason was documented on the eCRF.  ^e^ 28-day Follow-up visit was scheduled 28 days (+7 days) after discontinuation visit. ^f^ 12-lead ECGs (3 times, approximately 2 minutes apart) with central reading by a cardiologist, were performed as per planned schedule, at the following times: Screening; First dosing day (Day 1 Cycle 0) for dose escalation phase: pre-dose, 1, 2, 4, 6, 8, 10, 12, 24 hours (Day 2) and 48 hours (Day 3) post-dose; Day 1, Day 8 and Day 15, Cycle 1 (Multiple Dosing for all phases): pre-dose; First day of multiple dosing (Day 1 Cycle 1) for dose expansion and extension phases: 2 and 4 hours post-dose; Day 1, Cycle 2 (Multiple dosing for dose escalation and expansion phases): pre-dose, 1, 2, 4, 6, 8, 10, 12 and 24 hours (Day 2) post-dose; On Day 1 of each subsequent scheduled cycle visit: one assessment at any time during day; On occurrence of any cardiac AE; Unscheduled Visit if needed; Discontinuation visit. The timing and number of ECGs could be altered depending on the emerging PK and safety profile. ^g^ LVEF was using an echocardiogram or MUGA at Screening and every 12±1 weeks until discontinuation visit. Additional assessments could be conducted at the discretion of the investigator within every 12 weeks or during discontinuation visit if clinically indicated. For any patient who had at least one echocardiogram or MUGA that was considered abnormal by local assessment, the Sponsor was to collect all echocardiograms or MUGAs (obtained at Screening and all subsequent assessments) for the purpose of a central read. ^h^ Unless the patients withdrew consent, they were followed for AEs from the date of informed consent until 28 days after the last dose of study treatment. In the event of serious or study drug-related toxicities, the patient was followed until resolution or stabilization.  ^j^ Clinical laboratory tests were not required if acceptable screening were performed within seven days prior to administration of study drug, unless the patient’s clinical condition had changed significantly. If results of laboratory tests within seven days prior to administration of the study drug were not available, clinical laboratory test were performed at baseline visit before intake of the study drug. If needed, additional clinical laboratory tests could be performed for safety evaluation of patients.  ^k^ Hospitalization for Day 1, 2, and 3 Cycle 0 and Day 1, 2 Cycle 2 was required for PK blood sampling. Patients took the first dose of the study drug at the center; if the patient vomited the dose it should not be replaced but the time of vomiting should be captured in source document.  ^m^ Cycle 2  ^n^ After Cycle 3 Abbreviations: **AE**, adverse events; **ECG**, electrocardiogram; **MUGA**, multiple gated acquisition; **PK**, pharmacokinetic | | | | | | | | | | | | |

**Supplementary Table 3.** Effect of lazertinib on isolated perfused rabbit hearts at 750 ms, 500 ms, and 250 ms cycle lengths

| **750 ms cycle length** | | | | | | | | | |
| --- | --- | --- | --- | --- | --- | --- | --- | --- | --- |
| **Dose**  **(µM)** | **MAPD_30_**  **(ms)** | **MAPD_50_**  **(ms)** | **MAPD_90_**  **(ms)** | **BVR**  **(ms)** | **TRI**  **(ms)** | **QT**  **(ms)** | **QT_peak_ (ms)** | **T_peak_T_end_ (ms)** | **EAD**  **(#/min)** |
| 1 | -15 | -9 | -3 | 0.78 | 12 | -7 | -1 | -6 | 0 |
| 3 | 1 | 3 | 9 | 0.90 | 8 | 1 | -5 | 6 | 0 |
| 10 | -3 | 1 | 8 | -0.30 | 10 | -3 | -2 | 0 | 0 |
| 30 | -31 | -30 | -22 | 1.01 | 8 | -17 | -27 | 10 | 0 |
| **500 ms cycle length** | | | | | | | | | |
| 1 | -10 | -7 | -4 | -0.50 | 6 | 3 | 2 | -7 | 0 |
| 3 | 2 | 5 | 5 | -0.03 | 3 | 10 | 8 | -7 | 0 |
| 10 | -9 | -3 | 3 | -0.47 | 12 | 14 | 14 | -8 | 0 |
| 30 | -43 | -41 | -34 | -0.35 | 8 | 2 | -10 | 2 | 0 |
| **250 ms cycle length** | | | | | | | | | |
| 1 | 0 | 1 | 2 | -0.64 | 0 | 2 | 5 | -3 | 0 |
| 3 | 11 | 11 | 9 | -0.50 | 0 | 0 | 7 | -7 | 0 |
| 10 | -11 | -10 | -12 | -0.61 | 0 | 3 | 7 | -4 | 0 |
| 30 | -24 | -24 | -27 | -0.20 | 0 | 10 | 9 | 0 | 0 |
| **BVR**, beat-to-beat variability; **EAD**, early after-depolarization; **MAPD**, monophasic action potential duration; **MAPD­_30_**, MAPD at 30% repolarization; **MAPD­_50_,** MAPD at 50% repolarization; **MAPD_90_,** MAPD at 90% repolarization; **QT**, interval between the QRS complex and T wave; **TRI**, triangulation, reverse frequency-dependence, instability | | | | | | | | | |

**Supplementary Figure 1.** Effects of vehicle control^a^, lazertinib and E-4031^b^ on hERG potassium channel currents in hERG-transfected CHO cells

^a^ Normal Tyrode solution containing 0.3% DMSO

^b^ Positive control (100 nM E-4031)

^c^ Inhibition rates of all test articles except for vehicle are the compensated inhibition rates that are corrected by inhibition rate of vehicle control.

** P<0.01, compared with vehicle control using Dunnett’s test

†† P<0.01, compared with vehicle control using Student’s t-test

Abbreviations: **CHO**, Chinese hamster ovary; **DMSO**, dimethyl sulfoxide; **hERG**, human Ether-à-go-go-Related Gene; **N**, number of cells; **SEM**, standard error of mean

**Supplementary Figure 2.** Body temperature after single oral administration of 5, 10 and 20 mg/kg of lazertinib in conscious male beagle dogs

Data are expressed as mean ± standard error of mean (n=4)

**Supplementary Figure 3.** ΔQTcF values for 24 hours after single-dose (A) and at steady state (B)

Abbreviations: **ΔQTcF**, Change in QT intervals corrected with Fridericia’s formula from baseline; **ms**, millisecond; **h**, hours
